# Who can we reach and who can we keep? Predictors of intervention engagement and adherence in a cluster randomized controlled trial in South Africa

**DOI:** 10.1186/s12889-020-8357-x

**Published:** 2020-02-27

**Authors:** Stephan Rabie, Jason Bantjes, Sarah Gordon, Ellen Almirol, Jackie Stewart, Mark Tomlinson, Mary Jane Rotheram-Borus

**Affiliations:** 1 Institute for Life Course Health Research, Department of Global Health, Stellenbosch University, P O Box 241, Cape Town, 8000 South Africa; 2 Department of Psychology, Stellenbosch University, Private Bag X1, Matieland, 7602 South Africa; 3 Department of Psychiatry & Biobehavioral Sciences, Semel Institute, University of California Los Angeles, 10920 Wilshire Blvd., Suite 350, Los Angeles, California 90024 USA; 4 School of Nursing and Midwifery, Queens University, Belfast, UK

**Keywords:** Intervention implementation, Adherence, Engagement, At-risk men

## Abstract

**Background:**

Engaging and retaining young men in community-based interventions is highly challenging. The purpose of this study was to investigate the individual factors that predict intervention engagement and adherence in a sample of at-risk South African men.

**Methods:**

Baseline data were collected as a part of a cluster randomised control trial (RCT) situated in Khayelitsha and Mfuleni, two peri-urban settlements situated on the outskirts of Cape Town, South Africa. Neighbourhoods were randomised to one of three intervention conditions. We performed univariate descriptive statistics to report neighbourhood and individual socio-demographic factors, and ran multivariate models, adjusting for entry of study, to determine if high adherence and consistency of engagement with the intervention were associated with socio-behavioural demographics and risk behaviours, such as hazardous substance use, gangsterism, and criminal activity.

**Results:**

Total of 729 men were on average 22.5 years old (SD 2.8), with a mean of 10 years of education. More than half of the sample were single (94%), lived with their parents (66%) and had an income below ~$30 (52%). The overall mean of adherence is 0.41 (SD 0.24) and mean of consistency of engagement is 0.61 (SD 0.30). Our data indicated that completing more years of education, living with parents, and having higher socioeconomic status were significantly associated with higher rates of engagement and adherence. Men with a history of gang membership demonstrated higher levels of adherence and consistent engagement with the intervention, compared with other men who were recruited to the intervention. Crucially, our data show that young men with a history of substance use, and young men who report symptoms of depression and high levels of perceived stress are equally likely as other young men to adhere to the intervention and attend intervention sessions consistently.

**Conclusion:**

Our results may contribute to a better understanding of young men’s patterns of engagement and adherence to public health interventions. The results may have important implications for policy and practice, as they may be useful in planning more effective interventions and could potentially be used to predict which young men can be reached through community-based interventions.

**Trial registration:**

ClinicalTrials.gov registration, NCT02358226. Prospectively registered 24 November 2014.

## Background

Ensuring engagement and adherence to community-based public health interventions is highly challenging [1, 2] particularly when involving marginalised and underrepresented young men [3–5]. Engagement is defined as a multifaceted condition of behavioural, cognitive, and affective commitment to the intervention process [6], and signifies research participants’ continued involvement for the duration of a research study. Intervention adherence, in turn, refers to the extent to which research participants’ behaviour corresponds to the intervention condition assigned to them [7]; that is, the frequency with which participants attend the requisite number of contact sessions. Evidence suggests that intervention engagement and adherence is correlated with a variety of sociodemographic factors [2, 8–10] and intervention programme characteristics [2, 11, 12].

Considering the multiple risks faced by young men in resource-constrained communities, including substance use, HIV, interpersonal violence, and unemployment, engaging men in interventions addressing these public health concerns is imperative. Moreover, failure to achieve sufficient follow-up rates in these interventions threatens internal and external validity, reduces statistical power, wastes financial resources, and impedes researchers’ ability to identify subtle intervention responses within subgroups of participants [2]. Within this context, it is important to understand what individual factors predict adherence and engagement in these interventions in order to: [1] plan more effective interventions, [2] predict which young men can be reached through community-based public health interventions, and [3] work towards developing theories that help us understand patterns of adherence and engagement with interventions. The purpose of this study was to investigate the individual factors that predict intervention engagement and adherence.

### Individual characteristics associated with engagement and adherence

To the best of our knowledge, only a handful of studies have explored the individual sociodemographic correlates of engagement and adherence [1, 2, 11–14]. In their review of retention strategies employed in community-based clinical trials, Davis et al. [2] identified that engagement and adherence rates are negatively associated with participants’ age. That is, participants are more likely to drop out of community-based interventions if they are older. Gender and ethnicity have also been found to correlate with intervention retention, with evidence suggesting that in high-income countries, male participants from ethnic minorities are more likely to disengage from intervention programmes [1, 13]. Similarly, lower levels of education has consistently been found to influence engagement and adherence, with participants with fewer years of schooling more likely to drop-out of intervention programmes [2, 11, 12].

In addition to individual sociodemographic characteristics, intervention engagement is also influenced by participant clinical characteristics. For instance, participants with multiple health problems and patterns of erratic health care utilisation often demonstrate higher rates of attrition [2]. Emotional and psychosocial characteristics have also been found to be important predictors of intervention attrition, with some studies reporting that participants with higher levels of depression, hostility, and general psychological distress are more likely to drop out [14–16]. Similarly, one study reported that certain personality traits, particularly impulsiveness and novelty-seeking, are crucial correlates of attrition [16]. These findings suggest that there is a complex relationship between individual sociodemographic, clinical, and contextual factors that contribute to intervention engagement and adherence.

### Strategies to enhance retention

Within the context of the individual and clinical predictors of engagement and adherence, a number of studies have employed various strategies to enhance retention. Some have argued that knowledge of the individual characteristics that contribute to intervention attrition is crucial, as it allows for the design and development of programmes that are specifically targeted at individuals who are usually underrepresented, or difficult to access, in community-based interventions [12, 15].

Robinson et al. [12], in their systematic review of strategies used to improve participant retention, found the most common strategy that improves intervention engagement and adherence is the systematic methods of contact and scheduling procedures employed by project staff. Similarly, utilising communication strategies and maintaining between-assessment contacts (by for example, sending out newsletters, emails or telephone calls) has been found to significantly improve retention [11]. These strategies have been effectively employed in a range of interventions including mental health [17, 18], weight loss [19, 20], rare diseases [21], substance use [22], research involving minority ethnic groups [23, 24] and vulnerable groups such as the elderly [25], or people living with HIV [26].

Participants are also more likely to commit to a study long term if they understand both its importance and relevance [5]. The use of contact sessions (via home visits or phone calls) before or after informed consent to explain the study have been successfully used to enhance retention [5]. Huang et al. [27] recommends providing more clarity during the informed consent process to ensure that participants have a clear understanding of potential risks, benefits, rights, purpose and processes involved, which has been shown to increase retention. It is for this reason that well-trained staff with good interpersonal skills are critical factors in high retention studies [2]. Moreover, having staff who are embedded within the community and part of the same community where they are working or recruiting is associated with high retention rates [27].

Retention strategies can be labour intensive, especially for so-called hard-to-follow-up groups such as minority, rural, and low-income groups [9]. Within this context, the purpose of this study was to investigate individual factors that predict adherence and consistency of engagement with a community-based public health intervention in peri-urban settlements surrounding Cape Town, South Africa. The aims of the study were to: [1] describe the patterns of attendance at a public health intervention delivered over the 6 month period [2]; identify the sociodemographic factors associated with high levels of adherence and consistency of engagement in the intervention [3]; establish if at-risk young men (i.e. those with patterns of hazardous substance use, history of interpersonal violence / gangsterism / criminal activity, and patterns of high risk sexual behaviour) show different levels of adherence and consistency of engagement in the intervention.

## Methods

### Study setting

The data we report on were collected as part of a cluster randomised control trial (RCT) to test the efficacy of soccer and vocational training as contexts to deliver male-specific, HIV prevention programmes [28]. This intervention was staged in Khayelitsha and Mfuleni, two peri-urban settlements situated on the outskirts of Cape Town, South Africa. Khayelitsha has a conservatively estimated population of 420000 [28], and is one of the most impoverished areas in Cape Town, with a median annual household income of ~R20 000 (~US$1340), and half of its residents living in informal housing [29]. Khayelitsha is comprised of five major peri-urban settlements with both formal and informal housing. Mfuleni is located close to Khayelitsha, and is a relatively new peri-urban settlement, with an estimated population of 52300 [30]. Although reliable annual household income estimates are unavailable, the nature of housing and living conditions in Mfuleni is similar to those in Khayelitsha.

We identified 24 neighbourhoods of similar size (approximately 450–600 households) that was separated by buffer areas or at least 1 km of highways, railways, and rivers. Neighbourhoods were matched based on factors reflecting income (i.e. percentage of informal dwellings, availability of water and toilets on-site) and on density, ratio of dwellings to shebeens (informal bars), and access to day-care and healthcare clinics.

### Recruitment and randomization

Neighbourhoods were recruited in triplets, i.e. three neighbourhoods were enrolled at the same time. The assessment team then recruited the next triplet. In this cluster randomised controlled design, the UCLA team randomised neighbourhoods within matching triads and randomly assigned to one of three conditions: soccer league (SL), soccer league and vocational training (SL + V), and a control condition (CC). For this analyses, we will only be looking at those assigned to SL and SL + V conditions. There were a total of four waves of 150 recruited participants a month at the time of this analyses; recruitment for this study was ongoing at the time of this paper.

### Description of intervention

The SL group received soccer training for a 12-month period. On a weekly basis, participants attended 2 days of soccer practice, and one match day. The practices and matches were facilitated by soccer coaches, who were positive role models selected from the community and were trained in the foundational skills and theory common across evidence-based psychotherapeutic interventions and adolescent HIV prevention programs. The training included life skills in specific content areas (i.e., the core messages delivered during the intervention), including reducing alcohol/drug use, increasing HIV testing, optimizing the utilisation of healthcare facilities, fostering healthy daily routines, building friendship networks that are not based on shared risk behaviours, and managing money. The content areas were frequently rehearsed with role-plays during soccer practices so that the coaches could deliver the health messages on a regular basis.

For the SL + V condition, the SL condition described above was replicated for a six-month period. Thereafter, all young men were offered vocational training through two local organisations: Silulo Ulutho Technologies and Zenzele Training and Development. Through these organisations, young men were offered accredited training programmes in either computer courses, woodwork, or wielding. In addition, training was offered in a mentor-mentee environment, to enable participants to develop the necessary interpersonal skills required for employment.

Finally, the CC participants did not receive any intervention content, but routinely received flyers with picture stories regarding HIV prevention strategies and how to access these resources locally.

At the outset of the study, the fieldworkers and recruiters were blinded to intervention assignment.

### Participants

To be recruited into the study, participants were required to be unemployed young men; aged 18–29 years old; and in the preceding 2 months slept at least 4 nights per week in a dwelling within the neighbourhood they were recruited for at least 2 months prior to recruitment. In addition, participants were required to speak isiXhosa or English, and was not be under the influence of substances at time of recruitment. In keeping with the study design of a cluster RCT, all young men in a neighbourhood were assigned to the same condition. All young men assigned to the SL and SL + V were followed-up for the first 6 months. For this analysis, we focused primarily on the two intervention arms where soccer was implemented.

### Data collection

Baseline data were collected from a clustered sample of 729 young men who were assigned to either SL or SL + V conditions. Demographic characteristics included age, highest grade of school completed (years), relationship status, household monthly income, type of housing, presence of electricity in home, water access on the property, flushable toilets on premises, and presence of electrical source for cooking. Living with parents and partners were also recorded. Furthermore, we asked men about their chronic illnesses (e.g., HIV), social support, levels of gangsterism (i.e. number of arrests, number of prison sentences and gang membership), group violence involvement, and substance use [e.g. alcohol, marijuana (dagga), methaqualone (mandrax), and methamphetamine (tik)].

#### Depressive symptoms

The Center for Epidemiologic Studies Depression Scale (CES-D) was administered [31]. A cut-off of ≥16 was used to indicate depressed mood.

#### Perceived stress scale

The Perceived Stress Scale (PSS) was administered to measure the perception of stress [32]. A cut-off of 13 or higher was used to indicate high perceived stress.

*Substance Use* was self-reported if the participant used alcohol and/or used dagga, mandrax, or tik in the last 6 months. For alcohol consumption, binge drinking was asked if the participant consumed 6 drinks or more on one occasion on a daily or almost daily basis.

*Problematic drinking* was determined on whether the participant experienced heavy episodic drinking (six or more drinks in a single day) at least once a month over a specified timeframe, and responded yes to at least one of the following three questions: [1] Have close friends or relatives worried or complained about your drinking? [2]; Do you sometimes take a drink in the morning when you first get up? [3]; Has a friend or family member ever told you about things you said or did while you were drinking that you could not remember?

*Aggregate substance use* was calculated to measure the levels of substance by severity, i.e. alcohol, dagga, mandrax, and tik use. If a participant reported alcohol use, this was multiplied by 1; subsequently, if a participant reported dagga use, this response was multiplied by 2; and lastly, self-report use of mandrax or tik was multiplied by 3. Thereafter, these responses were summed up by each of these substances to create this aggregate variable of substance use.

#### Aggregate gangsterism

Similar to above, measures of gangsterism was calculated by severity, i.e. arrests, prison sentences, and gang memberships. If a participant reported being arrested, this was multiplied by 1; if a participant reported whether they were given a prison sentence, this response was multiplied by 2; and lastly, membership with a gang was multiplied by 3. Thereafter, these responses were summed up by each of these substances to create this aggregate variable of gangsterism.

Lastly, practice records were kept of the young men’s attendance at the weekly soccer practices over a period of 6 months. In both intervention arms, participants were expected to attend 72 soccer practises and matches over a period of 6 months. Rates and patterns of attendance at soccer practices were mapped over time, based on date at which participants were recruited into the intervention. The rates and patterns of practises were used to determine *intervention adherence* and *consistency of intervention engagement.*

#### Intervention adherence

A measure of extent of participation in soccer practices. The measure is an index (i.e. value from 0 to 1) that reflects the number of soccer practices attended (numerator) over the total number of scheduled practices over the observed period (denominator).

#### Consistency of intervention engagement

A measure of the regularity of attendance at soccer practices. The measure is an index (i.e. value from 0 to 1) which reflects the number of weeks during which at least 1 soccer practice was attended of the total number of weeks during which soccer practices were scheduled (numerator) compared to the total number of weeks during which soccer practices where scheduled over the observed period (denominator).

### Data analysis

Univariate descriptive analysis was used to describe the neighbourhood and individual sample socio-demographics, general health, social support, gangsterism, and substance use. Soccer attendance rates were summarized by month and graphed across time, based on when the intervention was initiated. A post-hoc analysis was done to determine if the level of adherence and consistency of engagement were associated with the time of entry into the intervention. Given these findings, we adjusted for entry of level for the study in the multivariate models to determine if higher indexes of adherence and/or consistency of engagement with the intervention were associated with socio-behavioural demographics and risk behaviours, such as hazardous substance use, gangsterism, and criminal activity. All data analysis was performed in R version 3.0.1. results are reported as adjusted odds ratios (aORs) and the significance level for all analysis was set to alpha = 0.05.

## Results

Table 1 summarizes the characteristics of the neighbourhoods as well as participants assessed at the start of the intervention. More than half reported informal dwellings or shacks (55%) among the 24 neighbourhoods randomised, and houses to sebeen ratio was 66:1, with an average of 13 shebeens within a neighbourhood. The mean age of the sample was 22.5 years (SD = 2.8), with an average of 10 years of education. The large majority of the sample was single (93.8%), had a monthly household income below R500 (~$33USD) (51.5%), lived in formal housing (63.4%) with access to electricity (99.2%), a toilet on premises (77.0%), electricity as fuel for cooking (88.6%), and lived with their parents (65.7%). As such, the majority of the participants came from low socioeconomic status households.

**Table 1:** Neighbourhood and individual characteristics of sample as assessed at the start of the intervention (*N* = 729)

	N or %
*Neighbourhoods characteristics*	
Total number of dwellings, i.e. brick and shack structures	21,877
Informal dwellings/shacks	11,572 (53%)
Houses:shebeen ratio	66:1
Informal bar/Shebeen (average, per neighbourhood)	13, SD 4.3
Day Care Centre/Creches (average, per neighbourhood)	2, SD 1.5
Spaza Shops (average, per neighbourhood)	6, SD 3.0
*Demographic characteristics*	
Age, mean (SD)	22.5 (2.8)
Highest education level, mean (SD)	10.4 (1.5)
Married	45 (6.2%)
Income above 500 Rand	353 (48.4%)
Formal housing	334 (63.4%)
Access to electricity	523 (99.2%)
Access to water source (home)	284 (53.9%)
Access to toilet (on premises)	406 (77.0%)
Main source of fuel for cooking (electric)	467 (88.6%)
Living with Parents	479 (65.7%)
Living with Partners	45 (6.2%)
*General Health*	
Chronic Illness	
Alcoholism	110 (67.1%)
HIV	25 (15.2%)
Depression Score, mean (SD)	15.9 (9.8)
Depressed Case, score ≥ 16	320 (43.9%)
Perceived stress scale, mean (SD)	14.4 (7.4)
Stress Case, score ≥ 13	214 (59.0%)
*Social support and Gangsterism*	
Number of close friends, mean (SD)	2.6 (1.7)
Gangsterism	
Number of arrests lifetime, mean (SD)	0.7 (1.3)
Number of prison sentences lifetime, mean (SD)	0.3 (0.6)
Gang membership	168 (23.0%)
Aggregate/gang variable, mean (SD)	2.5 (1.8)
Group violence	425 (58.3%)
Group violence involvement	311 (42.7%)
*Substance Use, in the past 3 months*	
Alcohol use	529 (72.6%)
Drink 6 or more (Daily)	7 (1.2%)
Problematic Drinker	116 (15.9%)
Marjiuana / Dagga	427 (75.7%)
Mandrax	121 (57.1%)
Methamephetamine / Tik	132 (18.1%)
Aggregate substance score, mean (SD)	2.8 (2.0)

Over two-thirds (67%) of the sample self-reported alcohol use as a chronic problem and approximately 44% reported depressed mood. Almost a quarter of the sample reported gang membership (23%); 60% had been a part of a group who was attacked; and 43% chose to get involved in a physical fight to support others. Three-fourths of the sample self-reported alcohol (73%) and dagga use (76%), while 57% reported mandrax use and 18% reported tik use.

**Fig. 1:**
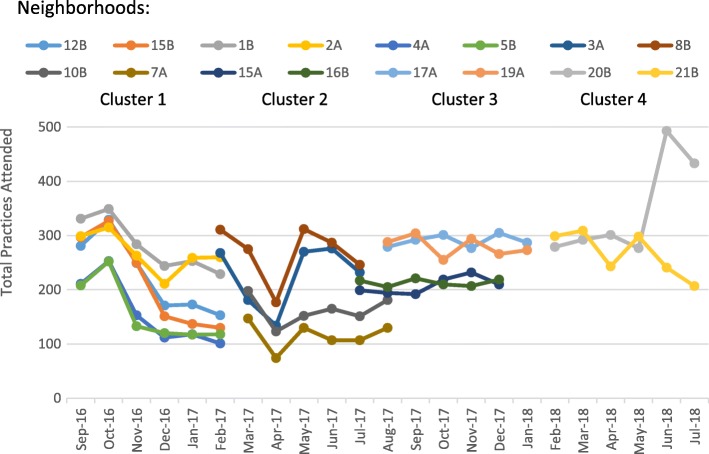
Attendance over time, by Start Month

Attendance rates over time are shown below in Fig. 1. The project scheduled an average of 240 practices per month, with each participant attending an average 1.3 practices per week. The soccer teams launched their intervention sessions at different time points, clustering into four main clusters. Each cluster ranged in attendance, from 193 (Cluster 2) to 306 (Cluster 4) practices per month. Within each cluster, a similar pattern of attendance was observed among teams. However, the date of entry into the intervention between the clusters may have affected the overall adherence and consistency. Because of these differing patterns of attendance by clusters over time, we performed a post-hoc analysis to establish the association between time of entry into the study and adherence and consistency of engagement.

Our analysis indicated that Clusters 3 (OR = 1.09, 95% CI: 1.01–1.13) and 4 (OR = 1.28, 95% CI: 1.19–1.38) had significantly higher adherence to the intervention, compared to Cluster 1.Clusters 3 (OR = 1.09, 95% CI: 1.04–1.15) and 4 (OR = 1.24, 95% CI: 1.14–1.36) had higher consistency when compared to Cluster 1. Cluster 2 had slightly lower odds of consistency when compared to Cluster 1 (OR = 0.89, 95% CI: 1.01–0.94). Considering these differences in adherence and attendance, the regression analyses controlled for date of entry into the intervention when comparing outcomes reflecting adherence and consistent attendance.

### Socio-demographic factors associated with adherence

The results for the unadjusted analyses are available as supplementary files Additional file 1. We performed multivariate models to identify associations between sociodemographic variables and adherence to intervention outcomes in (see Table 2). Our data indicated that married men and/or men living with their partners were less likely to adhere to the intervention than single men (*p* = 0.02), as were those living in informal housing (*p* < 0.01). However, completing more years of education (*p* < 0.01), living with parents (*p* = 0.05), living at a home with a water source on site (*p* < 0.01) and having a toilet on site (*p* < 0.01) were associated with higher rates of adherence. HIV-infected participants showed significantly lower adherence to the intervention, approaching significance (*p* = 0.05). No other associations were found between participant sociodemographic variables and adherence.

**Table 2:** Multivariate analysis of sociodemographic factors and risk behaviors associated with adherence ^✝^

	Estimated Adjusted Odds Ratio (aOR)		*P*-value
	aOR^a^	95% CI	
Married or lives with partner	0.92	0.85–0.99	0.02*
Education (years)	1.02	1.01–1.03	< 0.01*
Living with parents	1.03	1.01–1.08	0.05*
Living with partner	0.92	0.86–0.99	0.02*
Informal housing	0.88	0.84–0.93	< 0.01*
Water source on site	1.15	1.09–1.22	< 0.01*
Household toilet on site	1.18	1.12–1.24	< 0.01*
Gang Membership	1.04	1.01–1.09	0.02*

^✝^Analyses controlling for entry in study intervention; * *p* ≤ 0.05

### Socio-demographic factors associated with consistency of engagement

The results for the unadjusted analyses are available as supplementary files. We estimated multivariate models of the associations between sociodemographic variables and consistency of engagement in the intervention (see Table 3). Inconsistent attendance was associated with being a married man (*p* = 0.02), living with a partner (*p* = 0.02), and having informal housing (*p* < 0.01). However, consistent attendance was associated with greater years of education (*p* < 0.01), living with parents (*p* = 0.02), living at a home with water on site (*p* < 0.01) and having a toilet on the premises (*p* < 0.01). Having a monthly income greater than R2000 had a negative relationship with consistency in the unadjusted analysis (*p* = 0.04), however, not in the adjusted analysis (*p* = 0.26).

**Table 3:** Multivariate analysis of sociodemographic factors and risk behaviors associated with consistency of engagement^✝^

	Estimated Adjusted Odds Ratio (aOR)		P-value
	aOR^a^	95% CI	
Married or lives with partner	0.89	0.82–0.98	0.02*
Education (years)	1.02	1.01–1.04	< 0.01*
Living with parents	1.05	1.01–1.10	0.02*
Living with partner	0.90	0.82–0.99	0.02*
Informal housing	0.88	0.83–0.93	< 0.01*
Water source on site	1.18	1.10–1.26	< 0.01*
Household toilet on site	1.20	1.12–1.29	< 0.01*
Monthly household income > 2000	0.96	0.90–1.03	0.26
Gang Membership	1.06	1.01–1.12	0.02*
Aggregate gangsterism	1.02	1.01–1.04	0.02*

^✝^Analyses controlling for entry in study intervention; * *p* ≤ 0.05

### Public health and risk behaviours associated with adherence and consistency

Our multivariate analysis demonstrated that participants with gang membership were more adherent (*p* = 0.04) and consistent in attending the intervention sessions (*p* = 0.02). Aggregate gangsterism was also positively associated with consistency of engagement (*p* = 0.02). Symptoms of depression, levels of perceived stress, or substance use did not significantly predict poor adherence or consistency.

## Discussion

Our data demonstrate that there are clear associations between specific sociodemographic variables and both the level of adherence and of consistency in engagement with a community-based soccer intervention over a six-month period. In particular, our results suggest that young men are more likely to engage with this public health intervention and attend soccer practices regularly if they are single, have more years of schooling, live with their parents, and live in a house with access to a toilet on the premises.

It is not altogether surprising that young single men were more likely to engage in this time-consuming 6-month soccer intervention given that in South Africa, manhood is defined, both culturally and socially, by a man’s ability to provide economically for his family [33]. Fathers and men in committed relationships (i.e., married, living with partners etc.) are expected to assume the social responsibility of finding employment to ultimately become the primary provider for their families. Considering that unemployment was one of the inclusion criteria at baseline recruitment, our finding that single men are more likely to consistently attend and engage in our intervention may be a function of men in committed relationships being under social and cultural pressure to secure employment and provide for their families, and therefore less likely to attend soccer practises.

Our finding that intervention adherence and engagement was predicted by more years of schooling is consistent with previous research [2, 13, 34]. This is likely to be a function of the fact that individuals with higher levels of education have been primed to adhere and engage in intervention programmes, as exposure to more years of schooling is associated with healthy routines, structure, rules, and punctuality – all factors assumed to be related to adherence. Moreover, having access to a toilet on the premises in this study’s context is a proxy for socioeconomic status. Accordingly, our data suggests that young men are more likely to engage and attend regular soccer practices if they come from families with higher socioeconomic status. Although there is a paucity of literature on the association between socioeconomic status and community-based intervention adherence, research on college retention and engagement indicates that students from lower socioeconomic backgrounds are less likely to consistently attend and engage in college [35, 36].

Our results further show that participants living with their parents were more likely engage with and adhere to the intervention. Extant research has indicated that co-residence between parents and adult children is often characterised by increased parent-child involvement [37], and substantial financial, domestic, and financial support [38]. As such, living in their parents’ home may provide our participants with the necessary structure and external support and encouragement to consistently attend and engage in our soccer intervention.

Crucially, our data show that “at-risk men” (i.e., young men with a history of substance use, and young men who report symptoms of depression and high levels of perceived stress) are equally likely as other young men to adhere to the intervention and attend soccer practices consistently. Our data do not allow us to determine why “at-risk men” (i.e. those with a history of substance use, depression, high levels of stress) were as likely to adhere and engage with the intervention as other young men. This is, however, an interesting finding which needs to be more thoroughly explored in subsequent studies as it has important implications for designing public health interventions targeted at this hard to reach population.

Importantly, men with a history of gang membership demonstrated higher levels of adherence and consistent engagement with the intervention, compared with other men who were recruited to the intervention. The similarities in the characteristics between gangs and our intervention groups could provide a speculative explanation. Gangs are generally formed and maintained by members’ shared experience of alienation from traditional society [39], and offer a strong sense of community [40]. Similarly, the format in which our intervention is delivered could offer young men with a history of gang membership a network of social support, through which they develop a collective identity and establish a sense of belonging [41]. This finding has important implications, as it clearly demonstrates that interventions that are delivered in a group-setting and employ regular (twice weekly) soccer practices over a 6 month period for young men in low resource communities can be used to reach vulnerable and at-risk groups.

In particular, our data suggest that our intervention is especially appealing for young men with a history of gang membership. Moreover, men with histories of substance use, criminal activity, and those with symptoms of depression and stress are equally likely as other young men to adhere and consistently engage in our intervention. As such, community-based interventions that utilise sport and life skills to deliver content in a group setting appear to be a useful format to reach vulnerable and at-risk young men. Conversely, our data suggest that community-based interventions that utilise sport and life skills may not be useful in targeting young men who are married, have lower levels of education and low socioeconomic status. Alternative intervention strategies may therefore be more effective to ensure high levels of adherence and engagement. For instance, family-based interventions involving family members have been found to be effective to promote engagement in substance misuse treatment [42], and may be a potential strategy to engage young married men or men in committed relationship in this context.

### Limitations and recommendations

This study has several limitations. First, the substance use variables were only measured by self-reports. Future analyses should include rapid diagnostic testing for reliable measures of substance use. Moreover, the percentage of missing values for certain covariates included in the analyses could potentially create bias in the data. The current analysis did not investigate study characteristics as predictors of engagement and adherence. Future studies should explore the associations between individual characteristics, study characteristics, and intervention engagement and adherence.

## Conclusion

Engaging and retaining young men in community-based interventions is challenging. Our results suggest that single young men, who have more years of schooling, live with their parents, and who have higher socioeconomic status, are more likely to engage with our public health intervention. The results of this study may be useful in planning more effective interventions and could potentially be used to predict which young men can be reached through community-based interventions. More importantly, our results may contribute to a better understanding of young men’s patterns of engagement and adherence to public health interventions.

## Supplementary information

**Additional file 1.** The results for the unadjusted analyses

## Data Availability

The datasets used and/or analysed during the current study available from the corresponding author on reasonable request.

## Abbreviations

aORs: Estimated adkusted odds ratios
CC: Control condition
CES-D: The center for epidemiologic studies depression scale
CI: Confidence interval
HIV: Human immunodeficiency virus
OR: Odds ratio
PSS: Perceived stress scale
RCT: Randomised controlled trial
SL + V: Soccer league and vocational training
SL: Soccer league
