# Supplementary material for: Who can we reach and who can we keep? Predictors of intervention engagement and adherence in a cluster randomized controlled trial in South Africa
Source: BMC Public Health. 2020 Feb 27;20:275. doi: 10.1186/s12889-020-8357-x (PMC7045524; doi:10.1186/s12889-020-8357-x)
Supplement: Supplementary file 1 — Additional file 1. The results for the unadjusted analyses [file 12889_2020_8357_MOESM1_ESM.pdf]

Entry Date

```
  1   2   3   4 <NA>
278 184 181  91    0
```

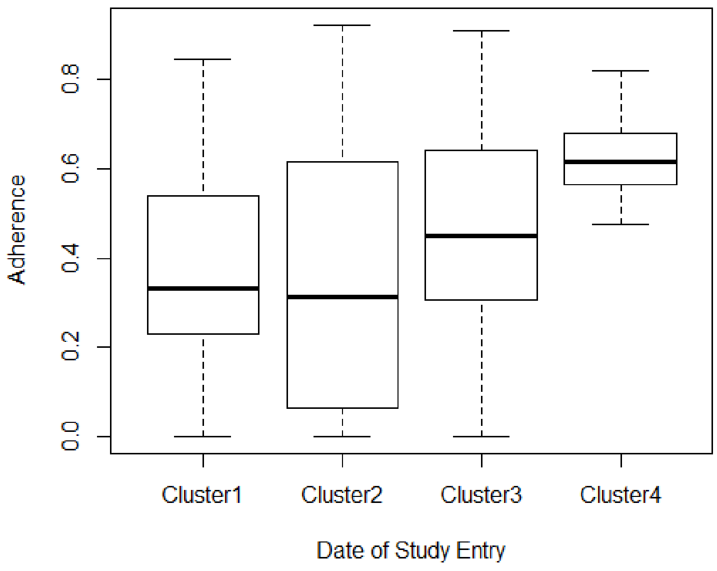

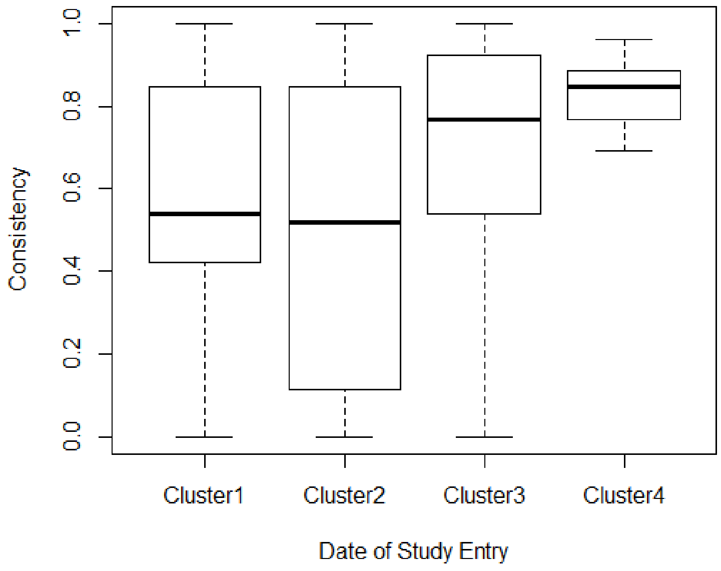


```
> cor(soc$adherence, soc$mstart, use = "complete.obs")
[1] 0.2303321

lm(formula = adherence ~ factor(mstart), data = soc)

Residuals:
     Min       1Q   Median       3Q      Max
-0.46168 -0.17495 -0.02579  0.17120  0.56868

Coefficients:
                Estimate Std. Error t value Pr(>|t|)
(Intercept)      0.38008    0.01404  27.075  < 2e-16
factor(mstart)2 -0.02568    0.02229  -1.152 0.249684
factor(mstart)3  0.08160    0.02248   3.630 0.000305 ***
factor(mstart)4  0.24528    0.03755   6.532 1.28e-10 ***
```

```
> cor(soc$consistency, soc$mstart, use = "complete.obs")
[1] 0.1803395

lm(formula = consistency ~ factor(mstart), data = soc)

Residuals:
     Min       1Q   Median       3Q      Max
-0.69665 -0.22126  0.00819  0.23896  0.50951

Coefficients:
                Estimate Std. Error t value Pr(>|t|)
(Intercept)      0.60719    0.01718  35.350  < 2e-16
factor(mstart)2 -0.11670    0.02728  -4.278 2.15e-05 ***
factor(mstart)3  0.08946    0.02751   3.252   0.0012 **
factor(mstart)4  0.21845    0.04595   4.754 2.43e-06 ***
```

Marital status

```
> table(soc$Marital_status, useNA="always")
   0    1 <NA>
 689   45    0
```

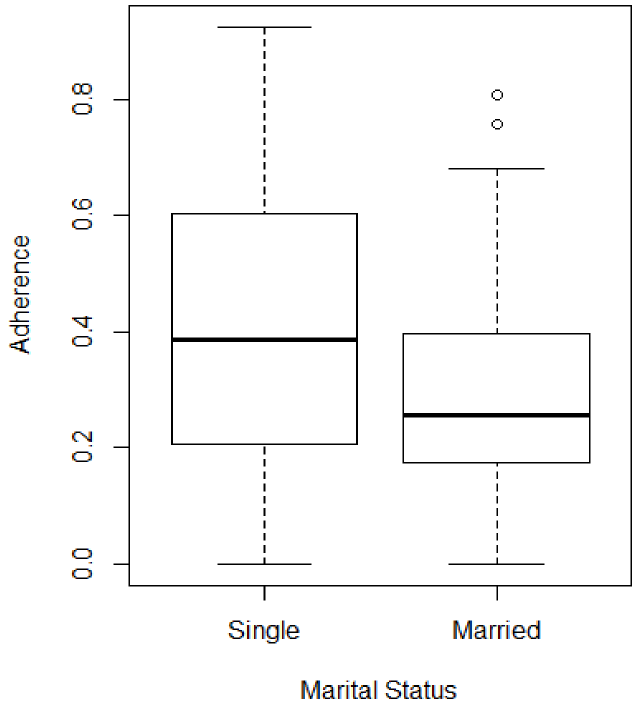

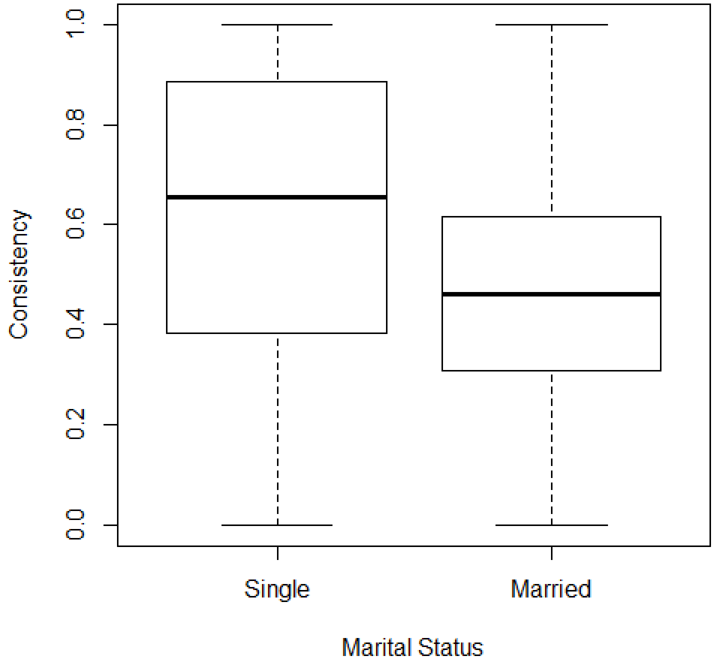


```
> cor(soc$adherence, soc$Marital_status, use="complete.obs")
[1] -0.09719881

sample estimates:
mean in group 0 mean in group 1
       0.4166968       0.3183761

lm(formula = adherence ~ factor(Marital_status) + factor(mstart
    data = soc)

Residuals:
     Min       1Q   Median       3Q      Max
-0.46362 -0.16875 -0.02672  0.16458  0.56303

Coefficients:
                        Estimate Std. Error t value Pr(>|t|)
(Intercept)              0.38720    0.01433  27.017  < 2e-16
factor(Marital_status)1 -0.08577    0.03729  -2.300 0.021753 *
```

```
> cor(soc$consistency, soc$Marital_status)
[1] -0.1002459

sample estimates:
mean in group 0 mean in group 1
      0.6214036       0.4963370

lm(formula = consistency ~ factor(Marital_status) + factor(
mstart),
    data = soc)

Residuals:
     Min       1Q   Median       3Q      Max
-0.69913 -0.19320  0.00229  0.22988  0.50229

Coefficients:
                  Estimate Std. Error t value Pr(>|t|)
(Intercept)        0.61628    0.01753  35.155  < 2e-16
(Marital_status)1 -0.10943    0.04561  -2.399  0.01670 *
```

Education

```
   Min. 1st Qu.  Median    Mean 3rd Qu.    Max.    NA's
   2.00   10.00   11.00   10.42   12.00   12.00       1
```

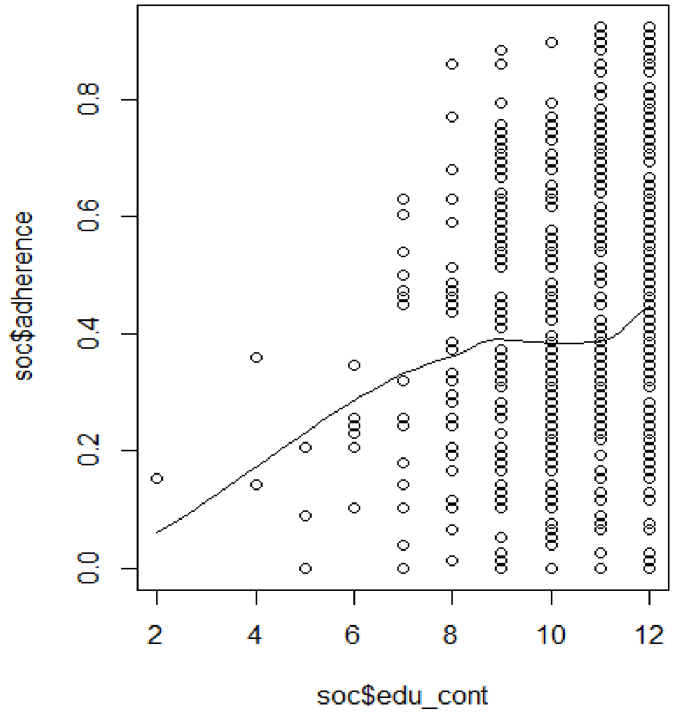

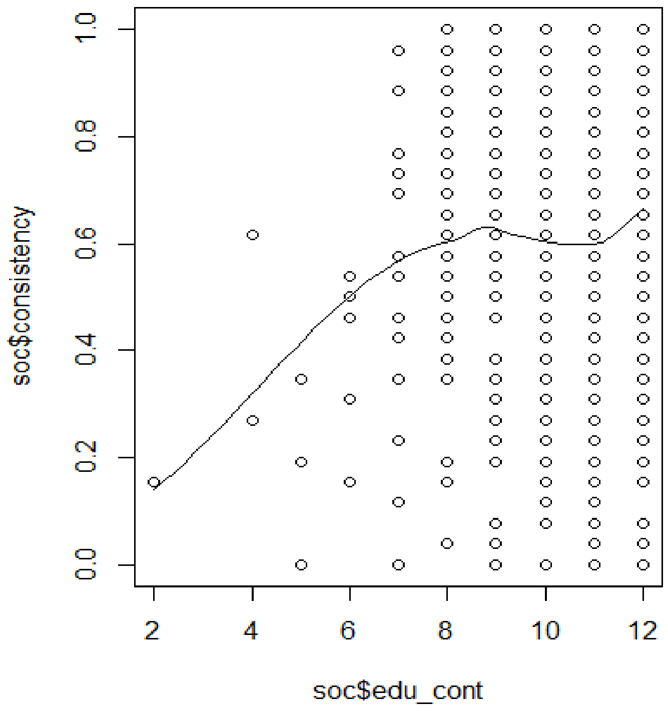


```
> cor(soc$adherence, soc$edu_cont, use="complete.obs")
[1] 0.163376

lm(formula = adherence ~ edu_cont + factor(mstart), data = soc

Residuals:
     Min       1Q   Median       3Q      Max
-0.49426 -0.17375 -0.02599  0.17559  0.56227

Coefficients:
                 Estimate Std. Error t value Pr(>|t|)
(Intercept)      0.154773   0.060115   2.575 0.010247
edu_cont         0.022092   0.005735   3.852 0.000128 ***
```

```
> cor(soc$consistency, soc$edu_cont, use="complete.obs")
[1] 0.1231079

lm(formula = consistency ~ edu_cont + factor(mstart), data
= soc)

Residuals:
     Min       1Q   Median       3Q      Max
-0.72771 -0.20100  0.01534  0.23948  0.54372

Coefficients:
                 Estimate Std. Error t value Pr(>|t|)
(Intercept)      0.392389   0.073873   5.312 1.48e-07
edu_cont         0.021062   0.007047   2.989  0.00290 **
```

Living with Partners

```
> table(soc$Partner.living.with.you2, useNA="always")
   1     2   <NA>
  45   592   97
```

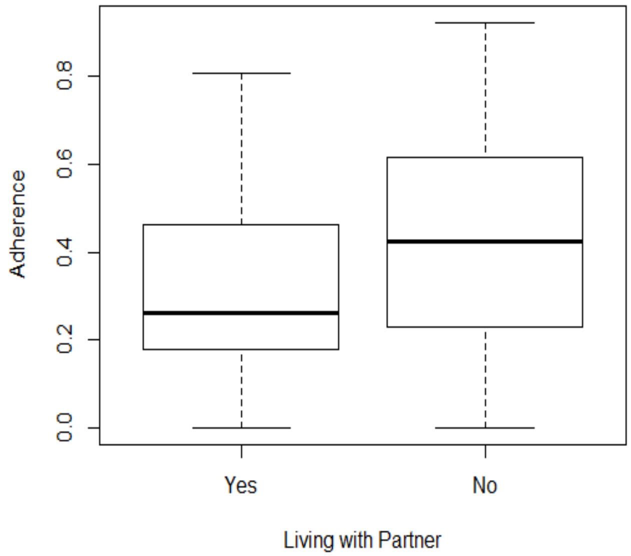

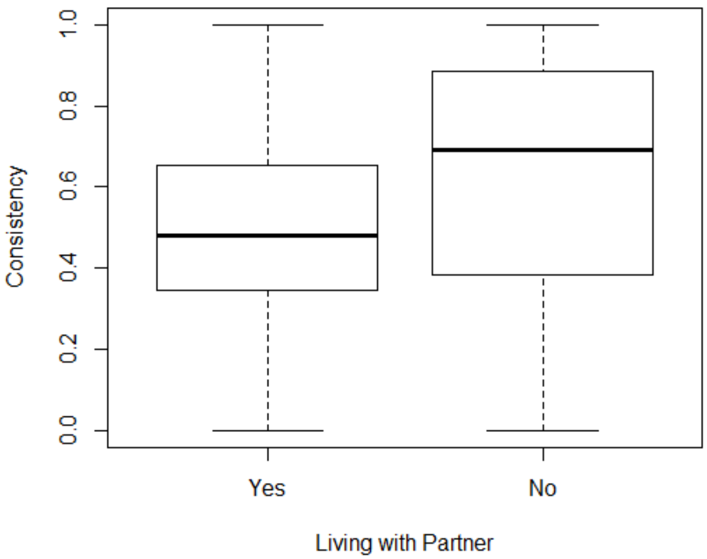


```
> cor(soc$adherence, soc$Partner.living.with.you”)
[1] 0.1007577

sample estimates:
mean in group 1 mean in group 2
      0.3183761       0.4131340

lm(formula = adherence ~ factor(Partner.living.with.you3)
+ factor(mstart), data = soc)

Residuals:
     Min       1Q   Median       3Q      Max
-0.46174 -0.16563 -0.02584  0.16646  0.57956

Coefficients:
                 Estimate Std. Error  t value Pr(>|t|)
(Intercept)      0.30719    0.03652   8.411 3.11e-16
(Partner.living3)0.08177    0.03693   2.214  0.02719*
```

```
> cor(soc$consistency, soc$Partner.living.with.you)
[1] 0.1002595

sample estimates:
mean in group 1 mean in group 2
      0.4963370       0.6187535

lm(formula = consistency ~ factor(Partner.living.with.you3)
+ factor(mstart), data = soc)

Residuals:
     Min       1Q   Median       3Q      Max
-0.69911 -0.19644  0.01499  0.22664  0.52217

Coefficients:
                      Estimate Std. Error t value Pr(>|t|)
(Intercept)           0.51434    0.04490  11.456  < 2e-16
(Partner.living3)2    0.10518    0.04540   2.317  0.02086 *
```

Formal housing

```
> table(soc$Housing.description, useNA="always")
   1     2   <NA>
 336   195   203
```

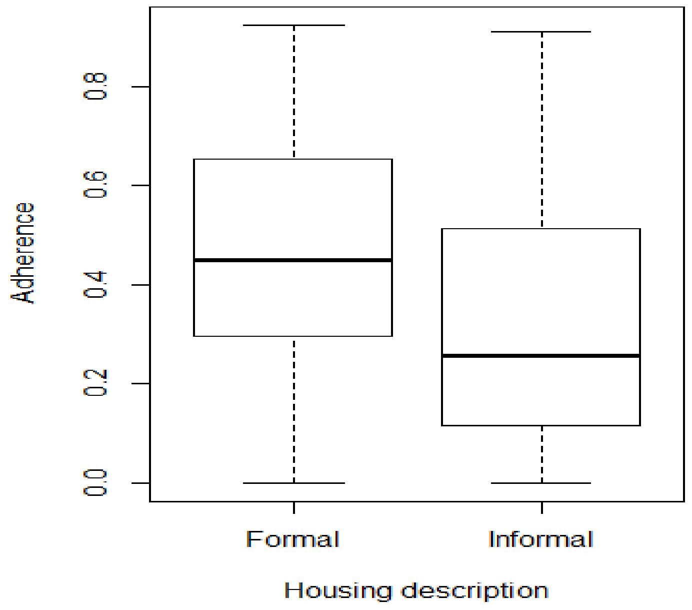

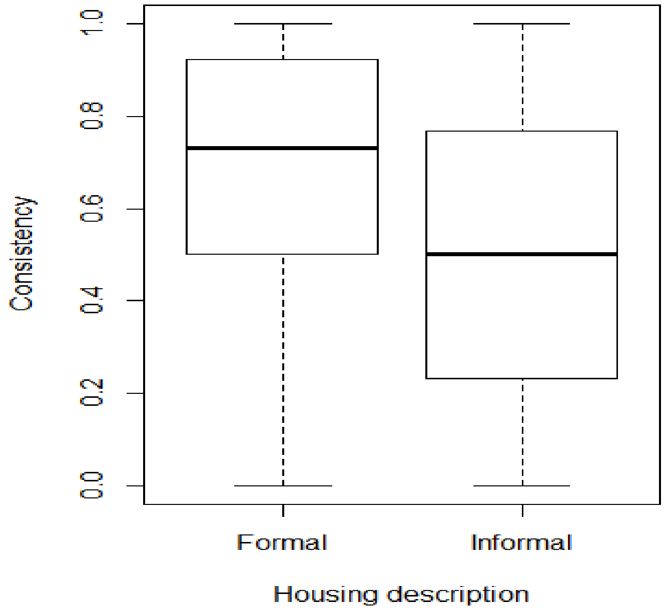


```
> cor(soc$adherence, soc$Housing.description)
[1] -0.2787248

sample estimates:
mean in group 1 mean in group 2
      0.4767927       0.3348045
lm(formula = adherence ~ factor(Housing.description)
+ factor(mstart), data = soc)

Residuals:
     Min       1Q   Median       3Q      Max
-0.47810 -0.16439 -0.01968  0.17418  0.57026

Coefficients:
                         Estimate Std. Error t value Pr(>|t|)
(Intercept)               0.42080    0.02234  18.833  < 2e-16
 (Housing.description)2 -0.12107    0.02384  -5.078 5.49e-07***
```

```
> cor(soc$consistency, soc$Housing.description)
[1] -0.2858479

sample estimates:
mean in group 1 mean in group 2
      0.6956975       0.5161833

lm(formula = consistency ~ factor(Housing.description) +
factor(mstart), data = soc)

Residuals:
     Min       1Q   Median       3Q      Max
-0.71454 -0.18925  0.01623  0.23382  0.55642

Coefficients:
              Estimate Std. Error t value Pr(>|t|)
(Intercept)    0.65079    0.02762  23.561  < 2e-16
(Housing)2    -0.13191    0.02947  -4.475 9.57e-06 ***
```

Water Source

```
> table(soc$Water.source)
  1    2    3 <NA>
286  133  112  203
```

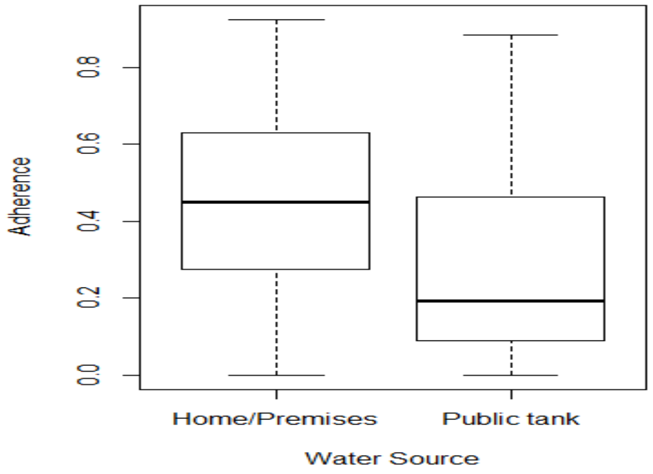

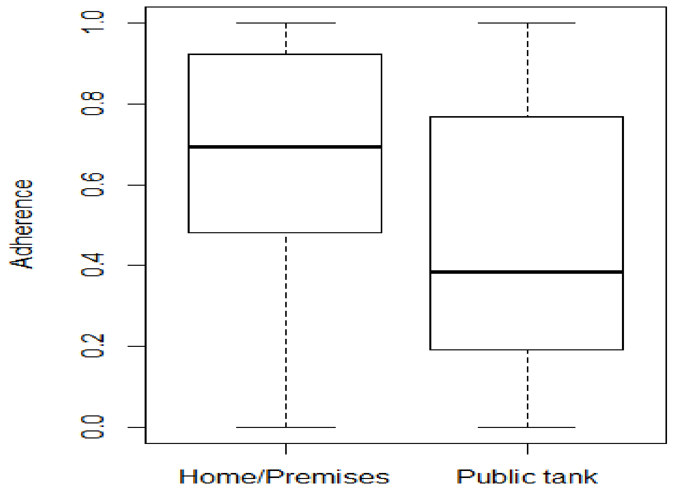


```
> cor(soc$adherence, soc$Water.source2)
[1] -0.3180569

sample estimates:
mean in group 1 mean in group 2
      0.4645530       0.2781339


lm(formula = adherence ~ factor(Water.source2) + factor(mstart),
    data = soc)

Residuals:
     Min       1Q   Median       3Q      Max
-0.46650 -0.15365 -0.02412  0.17261  0.60264

Coefficients:
                       Estimate Std. Error t value Pr(>|t|)
(Intercept)             0.41006    0.02138  19.180  < 2e-16
factor(Water.source2)2 -0.14204    0.02823  -5.031 6.96e-07***
```

```
> cor(soc$consistency, soc$Water.source2)
[1] -0.3303797

sample estimates:
mean in group 1 mean in group 2
      0.6800416       0.4451567

lm(formula = consistency ~ factor(Water.source2) + fact
or(mstart), data = soc)

Residuals:
     Min       1Q   Median       3Q      Max
-0.70216 -0.17157  0.02051  0.24098  0.59568

Coefficients:
                  Estimate Std. Error t value Pr(>|t|)
(Intercept)        0.64165    0.02637  24.332  < 2e-16
(Water.source2)2 -0.16238    0.03483  -4.663 4.07e-06***
```

Toilet

```
> table(soc$Water.source)
  1    2    3 <NA>
286  133  112  203
```

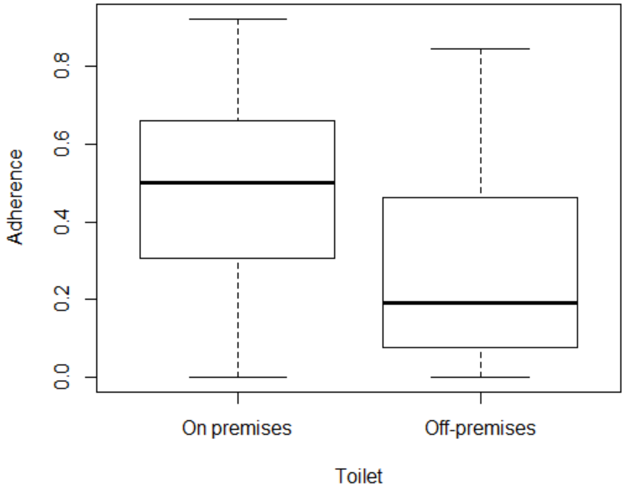

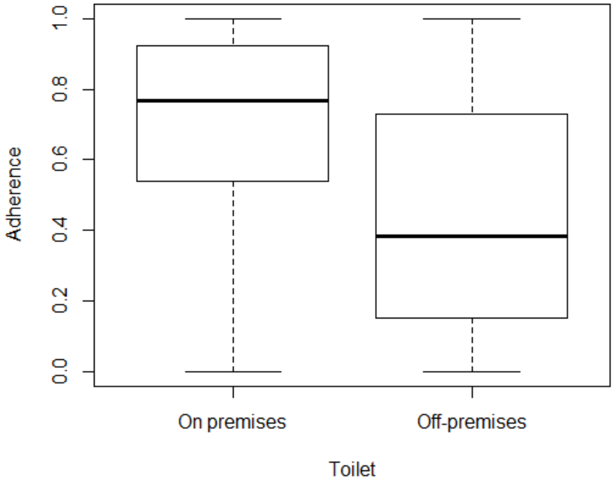


```
> cor(soc$adherence, soc$Water.source2)
[1] -0.3180569

sample estimates:
mean in group 1 mean in group 2
      0.4645530       0.2781339


lm(formula = adherence ~ factor(Household.toilet2)
+ factor(mstart), data = soc)

Residuals:
     Min       1Q   Median       3Q      Max
-0.46736 -0.15883 -0.02279  0.17322  0.56999

Coefficients:
                      Estimate Std. Error t value Pr(>|t|)
(Intercept)            0.42557    0.02161  19.689  < 2e-16
(Household.toilet2)2 -0.16734    0.02756  -6.073 2.58e-09***
```

```
> cor(soc$consistency, soc$Water.source2)
[1] -0.3303797

sample estimates:
mean in group 1 mean in group 2
      0.6800416       0.4451567

lm(formula = consistency ~ factor(Household.toilet2) +
factor(mstart), data = soc)

Residuals:
     Min       1Q   Median       3Q      Max
-0.70289 -0.16496  0.02051  0.22254  0.59880

Coefficients:
              Estimate Std. Error t value Pr(>|t|)
(Intercept)    0.65661    0.02677  24.526  < 2e-16
(Toilet2)2    -0.18396    0.03413  -5.390 1.11e-07 ***
```

Chronic Illness

```
  TB     Alcoholism     HIV    <NA>
   29     111            25     569
```

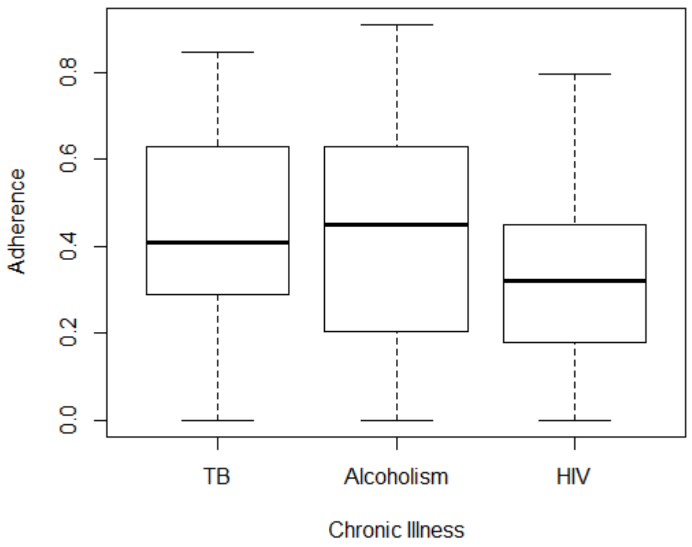

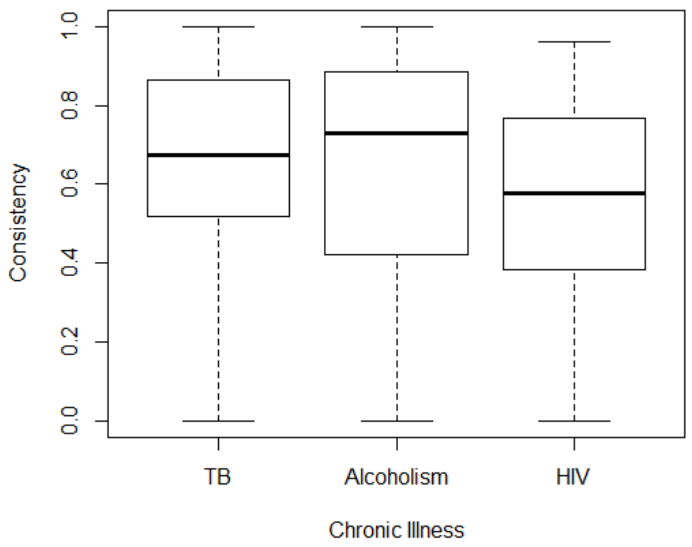


```
> cor(soc$adherence, soc$Chronic.illness)
[1] -0.09029262

lm(formula = adherence ~ factor(Chronic.illness) + fac
tor(mstart), data = soc)

Residuals:
     Min       1Q   Median       3Q      Max
-0.48646 -0.17572 -0.03592  0.16845  0.59450

Coefficients:
                  Estimate Std. Error t value Pr(>|t|)
(Intercept)        0.45941    0.05028   9.137 4.55e-16
(Chronic.illness)2 -0.04069   0.04892  -0.832  0.40682
(Chronic.illness)3 -0.12964   0.06669  -1.944  0.05380
.
```

```
> cor(soc$consistency, soc$Chronic.illness)
[1] -0.06371669

lm(formula = consistency ~ factor(Chronic.illness) + fac
tor(mstart), data = soc)

Residuals:
     Min       1Q   Median       3Q      Max
-0.73358 -0.18199 -0.00011  0.22796  0.60061

Coefficients:
                    Estimate Std. Error t value Pr(>|t|)
(Intercept)          0.70201    0.06101  11.506  < 2e-16
(Chronic.illness)2 -0.03307    0.05936  -0.557 0.578244
(Chronic.illness)3 -0.13540    0.08092  -1.673 0.096388
```

Gang Memberships

```
   1    2 <NA>
 168  566    0
```

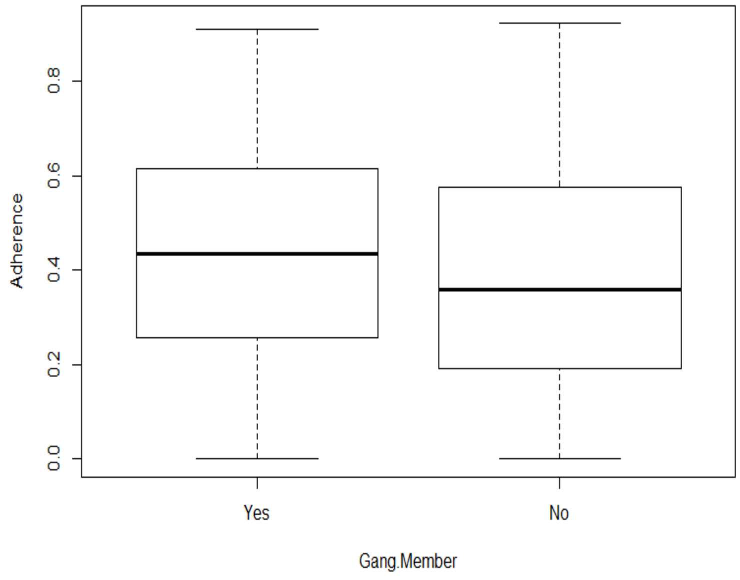

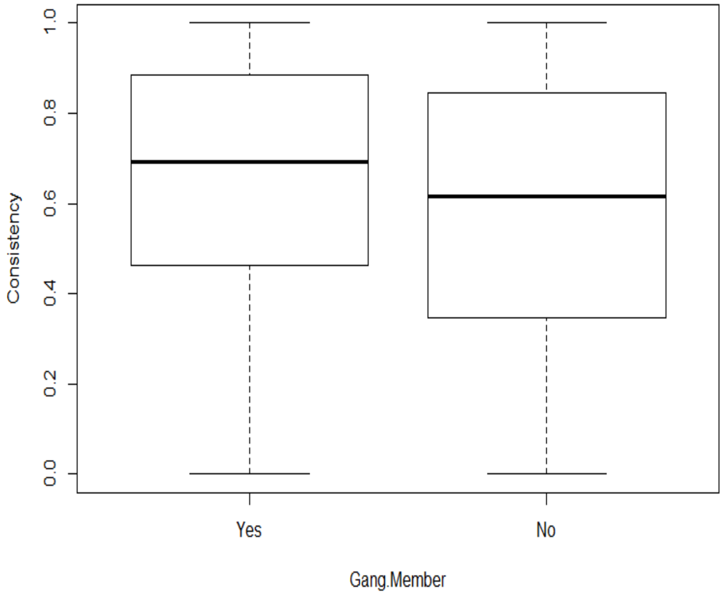


```
> cor(soc$adherence, soc$Gang.Member)
[1] -0.06553047

sample estimates:
mean in group 1 mean in group 2
      0.4395259       0.4018322

lm(formula = adherence ~ factor(Gang.Member) + factor(mstart),
    data = soc)

Residuals:
     Min       1Q   Median       3Q      Max
-0.49228 -0.16759 -0.02656  0.16756  0.57782

Coefficients:
                     Estimate Std. Error t value Pr(>|t|)
(Intercept)           0.41271    0.02144  19.246  < 2e-16
factor(Gang.Member)2 -0.04264    0.02122  -2.010 0.044865*
```

```
> cor(soc$consistency, soc$Gang.Member)
[1] -0.08273959

sample estimates:
mean in group 1 mean in group 2
      0.6586841       0.5999853

lm(formula = consistency ~ factor(Gang.Member) + factor(mst
art),
    data = soc)

Residuals:
     Min       1Q   Median       3Q      Max
-0.73929 -0.20863  0.01244  0.23194  0.52224

Coefficients:
                     Estimate Std. Error t value Pr(>|t|)
(Intercept)           0.65267    0.02622  24.896  < 2e-16
factor(Gang.Member)2 -0.05943    0.02594  -2.291  0.02227 *
```

Aggregate Gangsterism

```
   Min. 1st Qu.  Median    Mean 3rd Qu.    Max.    NA's
  1.000   1.000   1.000   2.494   4.000   6.000     459
```

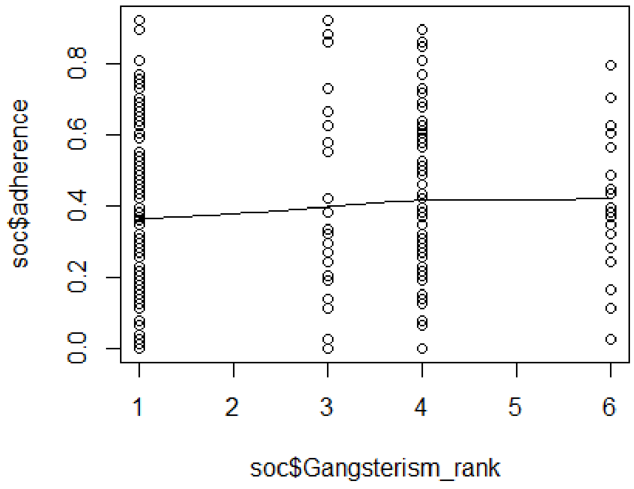

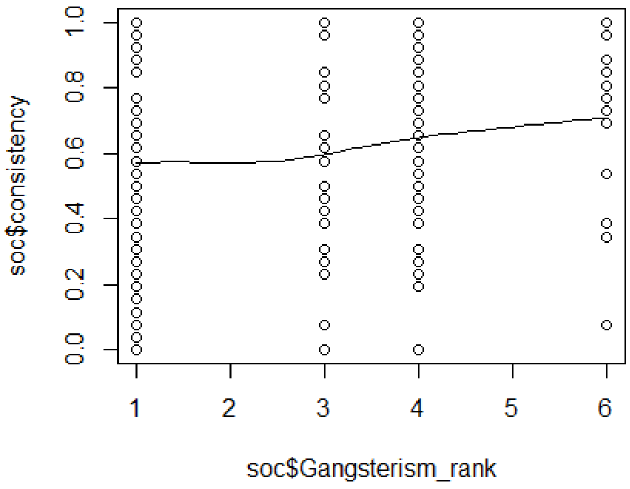


```
> cor(soc$adherence, soc$Gangsterism_rank)
[1] 0.07188372

lm(formula = adherence ~ Gangsterism_rank, data = soc)

Residuals:
     Min       1Q   Median       3Q      Max
-0.42513 -0.17803 -0.01778  0.17831  0.52709

Coefficients:
                 Estimate Std. Error t value Pr(>|t|)
(Intercept)      0.386265   0.025611  15.082   <2e-16
Gangsterism_rank 0.009717   0.008460   1.149    0.252
```

```
> cor(soc$consistency, soc$Gangsterism_rank)
[1] 0.1319719

lm(formula = consistency ~ Gangsterism_rank + factor(mstart
),
    data = soc)

Residuals:
    Min      1Q  Median      3Q     Max
-0.6305 -0.1802 -0.0145  0.2204  0.5610

Coefficients:
                  Estimate Std. Error t value Pr(>|t|)
(Intercept)       0.566399   0.035393  16.003  < 2e-16
Gangsterism_rank  0.023497   0.009857   2.384 0.017884 *
```
